# Read length versus Depth of Coverage for Viral Quasispecies Reconstruction

**DOI:** 10.1371/journal.pone.0047046

**Published:** 2012-10-03

**Authors:** Osvaldo Zagordi, Martin Däumer, Christian Beisel, Niko Beerenwinkel

**Affiliations:** 1 Institute of Medical Virology, University of Zurich, Zurich, Switzerland; 2 Institute of Immunology and Genetics, Kaiserslautern, Germany; 3 Department of Biosystems Science and Engineering, ETH Zurich, Basel, Switzerland; 4 SIB Swiss Institute of Bioinformatics, Basel, Switzerland; British Columbia Centre for Excellence in HIV/AIDS, Canada

## Abstract

Recent advancements of sequencing technology have opened up unprecedented opportunities in many application areas. Virus samples can now be sequenced efficiently with very deep coverage to infer the genetic diversity of the underlying virus populations. Several sequencing platforms with different underlying technologies and performance characteristics are available for viral diversity studies. Here, we investigate how the differences between two common platforms provided by 454/Roche and Illumina affect viral diversity estimation and the reconstruction of viral haplotypes. Using a mixture of ten HIV clones sequenced with both platforms and additional simulation experiments, we assessed the trade-off between sequencing coverage, read length, and error rate. For fixed costs, short Illumina reads can be generated at higher coverage and allow for detecting variants at lower frequencies. They can also be sufficient to assess the diversity of the sample if sequences are dissimilar enough, but, in general, assembly of full-length haplotypes is feasible only with the longer 454/Roche reads. The quantitative comparison highlights the advantages and disadvantages of both platforms and provides guidance for the design of viral diversity studies.

## Introduction

Next-generation sequencing (NGS) is changing dramatically our ability to analyze virus populations [1], [2]. With NGS, many viral genomes can be analyzed in parallel in a single sequencing experiment [3], and by using deep coverage, even rare viral variants can be detected in genetically heterogeneous populations. Deep sequencing of intra-host virus populations is becoming an important tool for studying viruses with a growing number of applications [4], including, for example, drug resistance [5], [6], [7], , immune escape [9], [10], and epidemiology [11], [12].

Most NGS-based studies assess viral diversity at each sequence position separately by inferring single-nucleotide variants (SNVs) from the read data. SNV calling is complicated by errors that can occur during sample preparation and sequencing, and statistical tests have been developed to distinguish technical errors from true biological SNVs [6], [13], [14], [15]. Since all NGS technologies amplify and read out individual DNA molecules [3], the co-occurrence of mutations, or phasing, can also be assessed provided that they are observed on the same read. By considering entire reads, rather than individual SNVs, error correction can be significantly improved, and the structure of the virus population, i.e., the set of all viral haplotype sequences and their frequencies, can be inferred over genomic regions as long as the average read length [13], [16]. The local haplotype inference problem is solved by clustering overlapping reads such that each cluster corresponds to one viral haplotype [17], [18], [19].

In highly diverse virus populations, such as RNA or single-stranded DNA viruses, mutations can be so frequent that they may be phased even if they are not observed on the same read. This global haplotype reconstruction problem becomes feasible if SNVs can be connected by a series of partially overlapping reads. It can be regarded as a sequence assembly problem from short reads, with the goal of reconstructing a viral quasispecies, i.e., a set of related sequences, rather than a single genome. Computational methods for viral quasispecies assembly include combinatorial optimization techniques [17], [20], [21], [22], [23] and generative probabilistic models [24], [25], [26].

SNV calling and local and global haplotype reconstruction assess viral genetic diversity at different spatial scales, ranging from single sites to the whole genome. Long-range haplotype reconstructions are more informative than short-range inference, because the linkage between mutations often has important phenotypic consequences. On the other hand, the statistical power to detect variation is highest for local haplotypes, and the computational complexity of haplotype assembly increases with the length of the genomic region. The optimal scale of diversity estimation also depends on the employed NGS platform and the read data it generates. Among other factors, NGS technologies differ in the number of reads they produce per run, the read length, the error pattern, and the cost per base [3]. However, it is unknown how sequencing platforms compare across the different viral diversity estimation tasks.

Here, we address this question and compare the two most commonly used NGS platforms for viral diversity estimation, namely 454/Roche pyrosequencing [27] and Illumina Genome Analyzer [28]. Previously, both platforms have been shown to exhibit similar mismatch error rates, while 454/Roche had an increased indel error rate in homopolymeric regions [29]. Instead of error profiles, we focus here on coverage and read length, two critical parameters for viral diversity estimation. Whereas 454/Roche produces longer reads, Illumina reaches higher coverage per run at lower costs, suggesting more power to detect low-frequency local variation with Illumina, but more power to assemble global haplotypes with 454/Roche data. We investigate this trade-off by analyzing a mixture of patient-derived viral clones that has been sequenced on both platforms and by simulated reads. We show how coverage, read length, and error rate jointly affect the performance of local and global haplotype inference. Our results provide guidance for the optimal choice of a NGS platform in viral diversity studies.

## Methods

### Experimental setup

Samples consisted of a mixture of PCR products from the *gag*/*pol* region of HIV-1 (positions 2253 to 3497 of HXB2 reference), obtained from plasma isolates of 10 infected patients and cloned into pCRII-TOPO vector. The isolates were collected as part of medically indicated HIV drug resistance tests and no additional samples were drawn for the purpose of this study. After processing, the PCR products had been routinely archived and were cloned for the purpose of quality control. All samples had been anonymized. A requirement for an ethics approval regarding projects as part of the quality control is not included in the statutes of the ethics commission of the state of Rhineland-Palatinate, Germany. The ten clones were mixed in different proportions, with intended relative frequencies between 0.1 and 50%. An aliquot of this sample was used as template in a PCR reaction, in order to study the impact of PCR amplification. These two samples were sequenced in parallel using 454/Roche FLX Titanium and Illumina Genome Analyzer 2, yielding a total of four experiments. Details of the sample preparation and 454/Roche sequencing have been reported elsewhere [16]. For Illumina sequencing, the 1.5 kb PCR amplicons were fragmented by sonication with a Bioruptor (Diagenode). Libraries were generated with the Illumina Genomic DNA sample preparation kit according to the manufacturer's instructions. Paired-end 36 cycle sequencing was done with the Illumina Genome Analyzer 2. In this work, we focus on a subset of the reads, namely those from the 252 bp region corresponding to protease amino acids 10 to 93 (nucleotides 2280 to 2531 on HXB2 reference). The mean sequence distance between the clones on this region is 7.5% (IQR 6–8.3%).

### Multiple sequence alignment

The tool ‘s2f.py’ included in the software package ShoRAH [30] was used to build a multiple sequence alignment (MSA) of the 454/Roche reads mapping the region corresponding to amino acids from 10 to 93 on the protease gene. Insertions were discarded as set in the default options. Only reads covering at least 80% of the region were retained. Illumina reads were aligned against the HXB2 reference sequence with the read mapper Novoalign (version 2.07.18, default parameter options, http://www.novocraft.com/). The paired ends were aligned independently. The output, in SAM format, was parsed with a custom script to estimate the diversity at each sequence position as the Shannon entropy 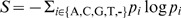, where *p*_i_ is the relative frequency of nucleotide *i* at this position. The MSA of the 35 bp region with the highest average entropy (around position 2451 on HXB2 for both non-PCR and PCR amplified samples) was extracted with the ShoRAH tool ‘bam2msa.py’.

### Direct frequency estimation

For each sample, we estimated the frequency of each clone directly by mapping all reads to the respective ten reference sequences and maintaining only the alignment of highest quality. The frequency estimate of a clone is then the proportion of reads mapping to it. We used the read mapper SMALT (version 0.6.3, word length 8, step size 2, http://www.sanger.ac.uk/resources/software/smalt/) for this purpose, because it can handle reads from both the Illumina and the 454/Roche platform. Frequency estimates can be obtained in this manner only if the sequences of the clones in the mixture are known in advance and if they are sufficiently different from each other, such that reads can be assigned uniquely. This was the case for our control experiment, but, in general, it does not hold for real-world applications. Here, we use the direct frequency estimates as a proxy for the real frequencies in the sample, which are unknown due to experimental inaccuracies in mixing the clones, and compare them to the estimates obtained from local haplotype reconstruction (see below).

### Local haplotype reconstruction

Local haplotype reconstruction aims at detecting viral variants in local windows of the MSA that are covered entirely by many reads and at correcting sequencing errors which would otherwise confound the inference. Using the ShoRAH program ‘diri_sampler’, we clustered the reads by sequence similarity, according to a probabilistic model for the generation of noisy reads from heterogeneous samples [18]. The predicted haplotype sequences are the cluster centroids (consensus sequences in each cluster) and their frequencies are the fractions of reads associated to each cluster. Probabilistic clustering was run for 10,000 iterations, including 8,000 for burn-in and 2,000 for sampling. The hyperparameter α was initially set to a value high enough to ensure a thorough exploration of the possible clustering configurations and then reduced during burn-in to a value where the configuration is almost stable, i.e, where cluster assignments of 90–95% of unique reads remain unchanged. The output includes a confidence value for each reconstructed haplotype. Haplotypes with confidence values smaller than 95% were discarded. Since we are analyzing a coding region, frameshift-causing insertions were removed and deletions were replaced by the consensus sequence. Local haplotype reconstruction was performed on the entire 252 bp region for the 454/Roche data, and on the 35 bp region of highest entropy for the Illumina reads.

### Simulation study

Reads were simulated from two mixtures of ten clones each under different conditions. The first set is based on the clones considered in the experiment described above, while the second set was designed to have lower diversity with a mean pairwise distance between haplotypes of 1.9% (IQR 1.2–2.4%). Reads were drawn in different numbers (10,000, 20,000, and 50,000) and at varying lengths (36, 75, and 150 bases) chosen to match the specifications of the Illumina platform over the years. Reads were drawn with equal probability from each clone resulting in 10% uniform frequencies per clone. The initial read positions were chosen with uniform distribution between the first position of the haplotype and the last one that allows the read to be entirely in the 252 bp region. Although it is possible to correct the sequencing error rate to some extent (see above), it is impossible to eliminate it completely. To take this limitation into account, we randomly added 0.01%, 0.05%, and 0.1% substitution errors per base to reproduce different levels of noise.

### Global haplotype reconstruction

Simulated reads were used as input to the global haplotype reconstruction procedure of ShoRAH using the programs ‘contain’, ‘mm.py’, and ‘freqEst’. Global haplotype inference was applied here only to the simulated data with a controlled sequencing error rate and hence ShoRAH was run without error correction. We considered the reads that are compatible with each other, i.e., that are identical on an overlapping region, and built the read graph, whose vertices correspond to reads and edges connect compatible reads. Haplotypes were reconstructed as paths in the read graph, such that all reads are explained by a minimal number of haplotypes. The relative frequencies of all inferred haplotypes are then estimated using an Expectation Maximization algorithm [2], [17].

## Results

We prepared a genetically diverse DNA sample by mixing ten HIV clones isolated from infected patients. One aliquot of this mixture was subject to PCR amplification. These two samples were sequenced in parallel using 454/Roche and Illumina Genome Analyzer, yielding a total of four experiments (Table 1). A total of 668 and 4,331 reads from 454/Roche sequencing were analyzed for the non-PCR amplified and PCR amplified sample, respectively. These numbers include all reads overlapping at least 80% of the amino acids 10 to 93 of the HIV-1 protease and represent the coverage of this region, which hosts the mutations associated with resistance to protease inhibitors. Segments of the reads falling outside of this region were discarded. The length of the remaining segments is 232±16 bases (mean ± std) and 236±18 bases for the two 454/Roche samples. Since we are dealing with a coding region, all insertions causing a frameshift were discarded. We did not detect any amino acid insertion or deletion. The Illumina experiments had a much higher throughput with more than one million reads mapped to the protease and local coverage of around 10,000 reads per base pair in the region further analyzed (Table 1).

**Table 1:** Summary statistics of sequencing experiments, read mapping, and error rates.

Platform	PCR amplification	Total reads	Reads mapped to protease (10–93)	Mapped read length (mean ± sd)	Reads included in the analysis	Error rate [%] (mean ± sd)
454/Roche	No	16,540	668	232±18	668	0.59±0.02
454/Roche	Yes	45,973	4,331	236±18	4,331	1.09±0.01
Illumina GA	No	12,559,696	1,505,619	36	11,835	0.17±0.01
Illumina GA	Yes	12,242,508	1,346,481	36	8,904	0.38±0.01

For all four experiments, the total number of reads obtained and those overlapping amino acids 10 to 93 of the protease are reported. All 454/Roche reads mapping to this region were used in the haplotype reconstruction. For the Illumina Genome Analyzer, only those mapping to the region of highest entropy were considered. The last column reports mean and standard deviation of the sequencing error rate (1 – θ, where the parameter θ is estimated during haplotype reconstruction).

**Figure 1:**
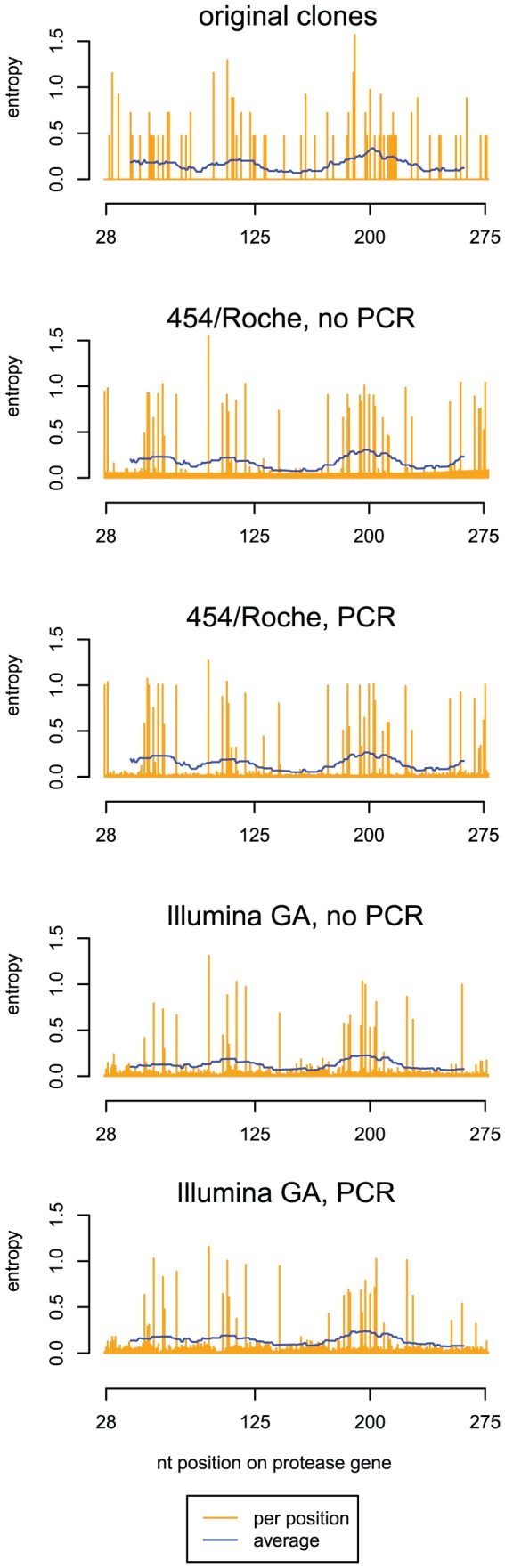
Diversity of the protease region measured on the multiple sequence alignments. The plot shows the Shannon entropy of each column of the multiple sequence alignment of all mapped reads (orange bars) and its moving average in a window of 35 bp (blue lines). Numbering of bases follows the nucleotide position on the protease, i.e., position 1 corresponds to position 2253 on HXB2. As a reference, the top subfigure shows the diversity of the mixture of the original ten clones assuming equal frequencies. The remaining subfigures refer to the four sequencing experiments using either 454/Roche or Illumina GA and PCR amplification or not.

Reads from the 454/Roche platform are long enough to display the diversity of the viral population on the entire 252 bp region. This is not the case for the Illumina data, as their 36 bases long reads are only able to cover a fraction of the amplicon. Nevertheless, by repeated local analyses on shifted smaller windows of the MSA one can find the genomic regions where the diversity is highest. We assessed the diversity of each sample by recording the fraction of different bases sequenced at each column of the alignment and computing the Shannon entropy of these distributions (Figure 1). Peaks in the entropy profile indicate polymorphic sites. The combined effects of low-frequency variants and sequencing errors contribute to a large number of sites with low but non-zero entropy. The entropy profiles were similar across all four experiments, with some differences due to the effect of PCR, which can disturb the clone frequencies, and different error profile of the two sequencing platforms, such as an elevated error rate of 454/Roche in homopolymeric regions (Figure 1). The entropy moving average was highest around nucleotide position 198 of the analyzed region corresponding to position 2451 on the HXB2 reference sequence.

### Local haplotype reconstruction

We performed local haplotype reconstruction on the four MSAs. For the Illumina data, we used the window of highest sequence diversity identified from the entropy profiles. We obtained 11,835 and 8,904 reads mapping to this region from the non-PCR amplified and PCR amplified sample, respectively. Local haplotype reconstruction was performed using the software ShoRAH [30], which infers local haplotypes from the multiple read alignment by correcting sequencing errors which otherwise would erroneously inflate the predicted diversity. In addition to the sequence compositions and frequencies of the haplotypes shaping the viral population, local haplotype inference also estimates the overall sequencing error rate, including both substitutions and deletions (insertions were discarded in the alignment step). For the non-PCR and PCR amplified samples from the 454/Roche platform, we estimated an error of 0.59±0.02 and 1.09±0.01% per base, respectively (Table 1). The Illumina platform showed less noise, with a sequencing error rate of 0.17±0.01 and 0.38±0.01%, respectively, for the non-PCR and PCR samples.

The performance of local haplotype reconstruction is assessed in terms of the number and frequency of haplotypes detected and their identity to the original sequences. A perfect haplotype reconstruction algorithm would detect exactly ten sequences, identical to the original clones. On the non-PCR amplified sample, we detected 13 haplotypes with 454/Roche, five of which are identical to five of the original clones (Table 2). With the Illumina platform, we detected ten haplotypes, nine of which each match perfectly one of the original sequences. For the PCR-amplified sample we reconstructed 30 and 10 haplotypes using 454/Roche and Illumina, respectively. In both cases, only six are perfectly matching one of the original clones. Both sensitivity (fraction of true haplotypes detected) and specificity (fraction of predicted haplotypes that are correct) are higher with the Illumina platform, for both the non-amplified and the amplified sample. This trend is also reflected in the per-clone frequency estimates compared with estimates obtained by direct mapping of the reads to the ten clones, which serve as the gold standard in this comparison (see Methods, Table 3). In particular, higher coverage and lower error rate, as achieved with Illumina, allowed for accurate calling of minor variants of frequencies as low as 0.2% that were not found in the 454/Roche data.

**Table 2:** Performance of local haplotype reconstruction.

Platform	PCR amplification	Reconstructed	TP	FP	FN	Sensitivity [%]	Specificity [%]
454/Roche	No	13	5	8	5	50	38
454/Roche	Yes	30	6	24	4	60	20
Illumina GA	No	10	9	1	1	90	90
Illumina GA	Yes	10	6	4	4	60	60

For all four experiments, we report the total number of predicted haplotypes (column Reconstructed), the number of correct haplotypes (true positives, TP), the number of reconstructed haplotypes that do not match any of the original clones (false positives, FP), and the number of missed haplotypes (false negatives, FN). This number is equal to 10 – TP, because ten is the total number of haplotypes present in the sample. Sensitivity is defined as TP/(TP+FN) and specificity as TP/(TP+FP). Local haplotype reconstruction was performed on the 252 bp region of the HIV *pol* gene coding for protease amino acids 10 to 93 for the 454/Roche data, and on the 35 bp subregion of highest entropy for the Illumina reads.

**Table 3:** Frequencies of all perfectly reconstructed haplotypes.

Platform	PCR amplification	Method	07-56681	07-54825	07-56951	08-59712	08-04134	08-01315	08-02659	08-57881	08-04512	Total
454/Roche	No	ShoRAH	10.6	14.1	14.1	13.9	4.9	—	—	—	—	57.6
454/Roche	No	Direct mapping	27.3	21.2	30.0	11.0	7.1	2.1	0.3	0.3	0.1	99.4
454/Roche	Yes	ShoRAH	3.6	15.7	22.0	11.4	7.0	0.3	—	—	—	60.0
454/Roche	Yes	Direct mapping	6.0	34.3	37.2	9.6	11.7	0.4	0.4	0.1	0.2	99.9
Illumina GA	No	ShoRAH	53.1	19.5	15.1	7.2	2.7	1.6	0.2	0.2	0.2	99.8
Illumina GA	No	Direct mapping	41.7	15.4	24.8	10.3	4.5	1.5	0.3	0.3	0.1	98.9
Illumina GA	Yes	ShoRAH	7.6	46.8	27.1	7.3	5.3	1.9	—	—	—	96.0
Illumina GA	Yes	Direct mapping	5.9	34.7	36.6	10.4	10.3	0.7	0.6	0.2	0.3	99.7

Reported are, for all four experiments, the relative frequencies in percent of the reconstructed haplotypes matching exactly one of the original clones (named 07-56681, …, 08-04512) as estimated by direct mapping and by ShoRAH. Undetected haplotypes are indicated by a dash (‘—’).

### Global haplotype reconstruction

Non-overlapping reads mapping to different positions of the genome can be assembled together to infer viral haplotypes over longer regions, provided that some conditions hold: consecutive reads must overlap significantly and host enough mutations to allow their unambiguous pairing. The first condition can be enforced by increasing coverage, whereas the second depends only on the diversity of the population to be analyzed. The presence of sequencing errors is a confounding factor that complicates inference. Thus, global haplotype reconstruction is expected to be easier with longer reads, larger diversity, and lower sequencing error rate. We assessed reconstruction performance as the proportion close (φ*_q_*), which is the fraction of reconstructed haplotypes that have at most *q* mismatches with respect to the original ones.

**Figure 2:**
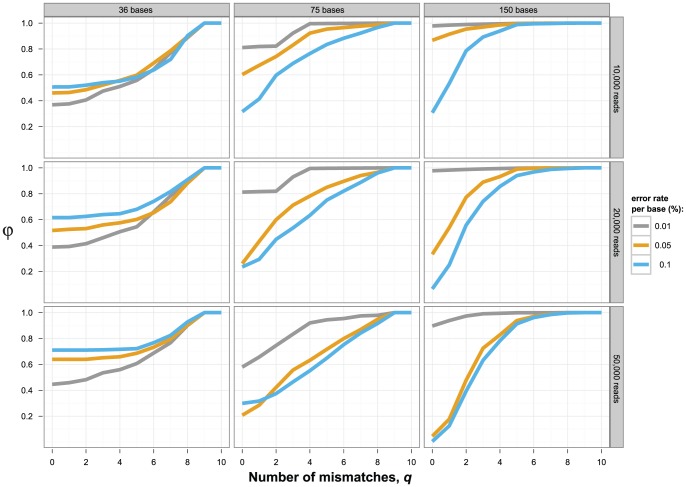
Global haplotype reconstruction at high diversity. The mean distance between clones of the underlying population was 7.5%. For global haplotype reconstruction, different conditions were tested, including varying read lengths (1st column: 36 bases, 2nd column: 75 bases, 3rd column: 150 bases), numbers of reads (1st row: 10,000, 2nd row: 20,000, 3rd row: 50,000), and sequencing error rates (grey: 0.01%, orange: 0.05%, blue: 0.1% per base). The y-axis reports reconstruction performance as the proportion close (φ*_q_*), defined as the fraction of reconstructed haplotypes that have at most *q* mismatches with respect to the original clones. The genomic region considered codes for amino acids 10 to 93 of the HIV protease.

**Figure 3:**
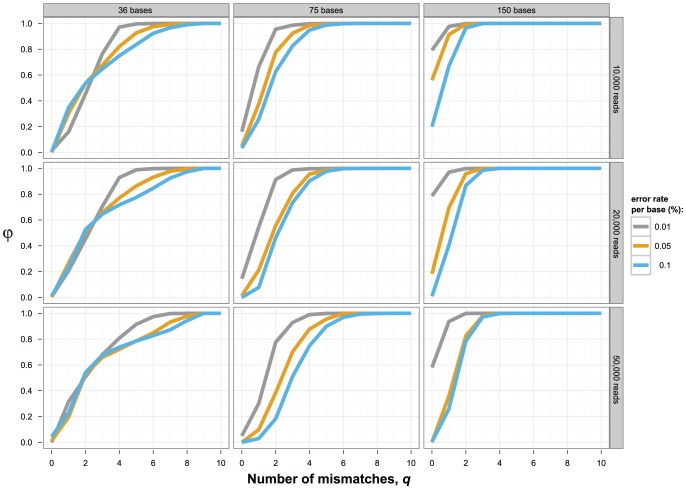
Global haplotype reconstruction at low diversity. Same as Figure 2, but the mean distance between clones is 1.9%.

The accuracy of global haplotype reconstruction was considerably higher when reads were drawn from the original clones which have a mean distance of 7.5% (Figure 2), than from the second haplotype set of lower diversity with mean distance 1.9% (Figure 3). Low population diversity renders perfect reconstruction (φ_0_ = 1) impossible, and the best one can achieve is reconstructing 80% of the haplotypes without mismatch. Coverage did not affect the performance of quasispecies assembly strongly, but sometimes higher coverage led to decreased performance, for example, for read length of 150 bases and error rate 0.01% in the high-diversity dataset, reflecting the limitations of global haplotype reconstruction (rows of Figures 2 and 3). By contrast, the read length has a strong impact on the inference of long haplotypes (columns of Figures 2 and 3). Even with a high level of diversity, 36 bases long reads are insufficient to infer haplotypes on a 252 bp long region reliably, regardless of the noise level or the coverage. However, the performance improves significantly when increasing the read length to 75 bases, and with the current reads of 150 bases, the haplotypes can be reconstructed with good accuracy between 60 and 100%, provided that errors are infrequent. A high error rate will decrease the reconstruction quality significantly, especially for longer reads of 75 and 150 bp.

## Discussion

We have presented a comparison of two sequencing platforms for the study of viral diversity highlighting the trade-offs between sequencing depth, sequencing errors, and read length. If the analysis is focused on a local region of the genome covered by the reads, then Illumina's higher accuracy and higher throughput enabling deep coverage are advantageous with respect to 454/Roche. In this case, haplotype reconstruction is both more sensitive and more specific for Illumina data. On the other hand, read length has a tremendous impact when one tries to match the diversity detected at sites more distant than the read length, and in this case, the 454/Roche platform has a clear advantage. Even the experimental Illumina datasets obtained from the highly diverse population analyzed here, do not allow for reliable reconstruction of the haplotypes. For example, with 36 bp long reads, regardless of the coverage and even assuming a low error rate, one can hardly reconstruct 50% of the population reliably (Figure 2). Thus, for long-range haplotype reconstruction in clinical samples, which often will display less diversity, read length appears to be the most critical factor.

Although both NGS technologies analyzed here have been improving rapidly in the last few years, their main distinctions remain. 454/Roche is still characterized by a higher indel error rate in homopolymeric regions. Illumina has a smaller total error rate, and a lower cost per sequenced base [31]. Both platforms increased their read length, with 454 now generating reads of average length 800 bp and Illumina of 150 bp, but their relative advantages and disadvantages are virtually unaltered. Of course, the performance of either platform can be boosted by increasing the coverage, but the sequencing error patterns remain a limiting factor. Importantly, increasing coverage is more cost-effective and less labor-intensive with Illumina than with 454/Roche.

To compare the relatively long-read 454/Roche sequencing platform with the short-read Illumina technology, we have considered a genomic region covered entirely by the long reads but not by the short reads. Since a head-to-head comparison is not possible, we have explored two approaches. First, we defined a local window of maximal average entropy in the hope of detecting the population diversity with local reconstruction methods from short reads there. This approach is particularly useful for diverse populations and although it will not result in the set of global haplotypes, it can be sufficient to estimate the population diversity, i.e., number and frequencies of clones. Second, we have attempted to assemble short reads into global haplotypes. This approach is statistically and computationally more challenging, but has the potential to recover all full-length haplotype sequences.

In dealing with NGS data, one has to take into account sequencing errors. Without a proper treatment, they would artificially inflate the estimated diversity. The approach presented here uses clustering of reads as a method to correct errors. Further measures would be to take quality scores into account, or to correct for strand bias. Variants that are observed prevalently on one strand are more likely to be artifacts than real biological variants [15]. The improved results obtained for the non-PCR amplified samples show that an additional source of noise is given by the PCR amplification, which can contribute in different ways to inflate and distort the observed diversity. Amplification efficiency can vary among different haplotypes, leading to an amplification bias. Moreover, PCR can introduce artificial variants into the sample by point mutations and, to a much larger extent, by recombination [16]. These *in vitro* chimera resulted in a larger number of false positives for 454/Roche than for Illumina (Table 2), because recombination is more likely to occur and to be detected in longer reads. Carefully chosen PCR conditions can minimize the impact of these artifacts [32].

For global haplotype reconstruction, we employed a combinatorial inference algorithm based on the read graph. This approach can easily generate recombinant sequences that are not part of the true underlying population, especially if diversity is low and not all read errors have been corrected. Such artificial *in silico* chimera are responsible for a large number of false positives in global haplotype reconstruction at deep coverage and might explain the decreasing global reconstruction performance with increasing coverage in some situations. Global haplotype inference may be improved by using alternative methods [20], [24], [26], [33], or by exploiting paired-end reads to phase variants detected at large genomic distances. The results presented here are subject to the specific limitations of ShoRAH's reconstruction algorithm. Other computational tools, including also improved error correction [34], might perform better under some circumstances, but the general limitation observed in this study will remain. Future studies are needed to delineate the feasibility of global haplotype reconstruction in terms of the underlying population diversity, the employed sequencing technology and parameters, and the computational strategy for haplotype inference.

The ability to detect and reconstruct diversity improves with decreasing sequencing error rate and with increasing number of polymorphic sites. As a consequence, for any given level of viral diversity in the sample, sequencing a longer region will result in better diversity estimates, for a given error rate. Since the diversity is usually unknown in advance, it is generally impossible to determine a priori the expected performance of a specific platform in reconstructing the viral population. We have highlighted and quantified here the trade-off between read length and depth of coverage, namely higher accuracy in global haplotype reconstruction with long reads versus improved sensitivity and specificity in local haplotype reconstruction, especially for low-frequency variants, with deep coverage.
